# Numerical Simulation and Experimental Study of Millisecond Percussion Drilling in Titanium Alloy

**DOI:** 10.3390/ma18153719

**Published:** 2025-08-07

**Authors:** Liang Wang, Long Xu, Changjian Wu, Yefei Rong, Kaibo Xia

**Affiliations:** 1 Faculty of Mechanical and Materials Engineering, Huaiyin Institute of Technology, Huaian 223003, China; wangliang@hyit.edu.cn (L.W.); jianjian56791116@163.com (C.W.); ryfxh96@163.com (Y.R.); 2 School of Mechanical Engineering, Jiangsu University, Zhenjiang 212013, China; xiakaibo@ujs.edu.cn

**Keywords:** titanium alloy, laser drilling, millisecond laser, numerical simulation, experimental verification

## Abstract

This study addresses the challenge of drilling film-cooling holes in the turbine blades of aircraft engines. Titanium alloy TC4 was selected as the experimental material. The laser-drilling process was simulated with ANSYS to determine optimal parameters, which were subsequently applied in machining trials. An impact-drilling method was then used to evaluate how pulse width, pulse energy, and pulse count affect micro-hole entrance and exit diameters, taper, and roundness. Simulations revealed that pulse energy and pulse count predominantly govern entrance and exit diameters, whereas pulse count and pulse width exert a stronger influence on taper. Experiments confirmed that entrance and exit diameters increased as pulse energy rose from 2.0 J to 2.8 J; taper increased as pulse width widened from 0.6 ms to 1.4 ms; and entrance diameter, exit diameter, and taper all grew as pulse count rose from 40 to 60. Pulse width and pulse count also significantly affected hole roundness.

## 1. Introduction

Aircraft-engine performance is strongly influenced by the film-cooling holes in turbine blades. Machining has long been challenged by their small diameter and large quantity. Drilling precision is greatly compromised by the high aspect ratio and complex spatial angles, and traditional methods are therefore considered inadequate to meet the required tolerances. These limitations are effectively addressed by laser machining, owing to its high efficiency and precision.

Titanium alloys are notoriously difficult to machine; however, their high specific strength and low thermal conductivity make them indispensable for aerospace and other high-performance applications [1]. A number of researchers have studied them. Wang Jiajia et al. [2] performed laser drilling experiments on titanium alloys using a water-assisted laser scanning method and established how each processing parameter affects hole diameter and taper. Liu Dan et al. [3] programmed in APDL within ANSYS to simulate the temperature field during the drilling process; by combining simulation and experiments, they compared the influence of laser process parameters on drilling quality and systematically summarized the general rules of nanosecond laser drilling of titanium alloys, with the aim of optimizing parameters and improving hole quality. Hou Hongling et al. [4] used ANSYS finite-element software to simulate the initial perforation and subsequent cutting stages in laser cutting, obtaining the reasonable perforation time for titanium alloy specimens of a given thickness, the propagation characteristics of temperature within the plate during cutting, and the temperature-field distributions at different cutting speeds. P. Deepu et al. investigated the morphology and surface integrity of femtosecond-laser-drilled microholes in titanium alloys. Through experiments, they examined the effects of laser parameters and scanning strategies on microhole characteristics and identified optimized parameters, including a laser energy density of 1.90 J/cm^2^, a pulse repetition frequency of 20 kHz, and a concentric-circle scanning strategy [5]. Satish Namdev et al. optimized the exit hole diameter of Ti-6Al-4V superalloy via laser drilling, validated their model using response surface methodology, and employed a genetic algorithm to optimize drilling parameters, thereby improving machining efficiency and hole quality [6]. In this paper, a two-dimensional nonlinear transient numerical model is established to study the effects of laser parameters on hole geometry and thermal effects during micro-drilling of titanium alloys, providing theoretical support for the practical application of laser micro-drilling technology [7]. Liang et al. used simulations to explore molten-pool behaviour during nanosecond laser machining of titanium alloys based on spiral scanning and its impact on the material removal mechanism, finding that unit energy density is the primary factor influencing material removal and revealing how temperature distribution affects the thickness of the recast layer [8]. Although laser machining offers many advantages, its thermal effects still lead to numerous defects in drilled holes; at present, producing high-quality microholes in difficult-to-machine materials remains a significant technical challenge [9,10,11].

Current research on titanium alloys primarily focuses on laser welding, laser cutting, and laser additive manufacturing [12,13,14]. In contrast, studies on laser drilling remain limited, and finite-element analyses of titanium alloys are even rarer. In this paper, we analyze laser drilling of titanium alloys using a combined simulation and experimental approach. First, ANSYS is used to simulate millisecond laser drilling. This simulation assesses how laser parameters affect microhole geometry (inlet diameter, outlet diameter, and taper) and the evolution of the temperature field. Second, experiments are conducted to examine how laser parameters influence microhole diameter, roundness, and taper. The experimental results are then compared with the simulation results. This work aims to improve understanding of how variations in laser parameters during millisecond laser drilling impact microhole diameter, roundness, and taper. It also provides a reference for subsequent investigations into the machining of film-cooling holes in titanium alloys.

## 2. Experimental Details

### 2.1. Material

Titanium alloy TC4(Baoji Titanium Industry Company Limited, Baoji, China), widely used in aircraft engines because of its unique properties, was selected as the experimental material. A 3 mm-thick, 30 mm-diameter disc was prepared. The chemical composition of titanium alloy TC4 is listed in Table 1.

### 2.2. Setup of the ANSYS

High complexity is encountered when simulating laser drilling. Therefore, the model was simplified by considering only the temperature field along the optical axis and at the interaction interface. The problem was thereby reduced to a two-dimensional model, markedly shortening computation time. The resulting 2D finite-element model measured 2 mm in length and 3 mm in width. During mesh generation, computational accuracy and cost were balanced, and a square mesh was adopted. More than five nodes were placed across the laser-spot diameter to ensure accuracy. PLANE55 four-node quadrilateral elements with a size of 2 μm were used, yielding a total of 15,251 nodes.

During laser drilling, the thermophysical properties of the titanium alloy—particularly thermal conductivity, specific heat capacity, and latent heat of phase change—strongly influence the temperature field. Throughout a phase change, temperature can rise from ambient conditions to several thousand degrees Celsius, and these properties vary continuously with temperature. Because the temperature-field simulation is nonlinear, temperature-dependent thermophysical data are essential. To simplify the heat-transfer model, all thermophysical properties—except thermal conductivity—were assumed constant. The absorption coefficient, density, and specific heat capacity were fixed, and latent heat was neglected. Titanium alloy TC4 was selected, with a melting point of 1668 °C, a boiling point of 3287 °C, and a density of 4.51 × 10^3^ kg m^−3^. The thermophysical parameters of TC4 are listed in Table 2.

**Figure 1:**
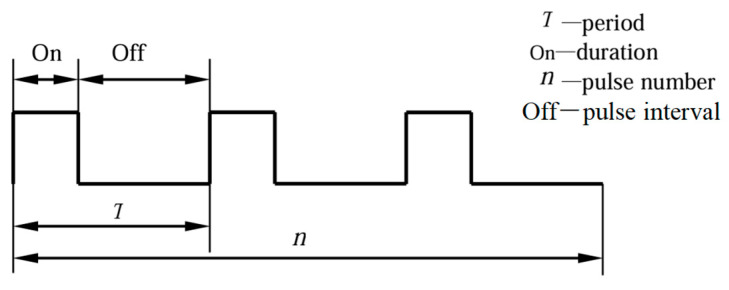
Pulse schematics.

(1) The experiment uses a pulsed laser, so the pulsed laser mode is selected for the simulation. The pulsed laser schematic is shown in Figure 1.

(2) Since the laser source used in the experimental part of this paper is characterized by a Gaussian distribution, a Gaussian heat source is used in the simulation, and the expression for the beam heat flux density is shown in Equation (1) [15] as follows:

(1) $$q=\frac{2AP}{\pi {\omega_{0}}^{2}}\exp(\frac{-2r^{2}}{{\omega_{0}}^{2}})=\frac{2A\cdot puls-one}{dura\cdot \pi {\omega_{0}}^{2}}\exp(\frac{-2r^{2}}{{\omega_{0}}^{2}})$$

where q is the heat flow density in the cross-section of the laser beam; A is the absorption rate of laser light by the material; P is the laser power; ω_0_ is the spot radius; r is the distance from a point on the surface of the material to the centre of the spot in the horizontal direction; puls-one is the single pulse energy of the laser; and dura is the pulse width. The simulation uses the life and death cell method to kill the lattice whose temperature reaches a set threshold.

(3) Initial condition: The initial temperature of the workpiece is the room temperature (25 °C), and it is spatially uniform throughout the workpiece. Boundary conditions: Laser drilling is extremely fast and acts on a very small spot, so the heat–affected zone is limited; therefore, the material can be regarded as an infinite medium. The side and bottom surfaces of the specimen are subjected to no thermal load (no heat flux) and are modelled as adiabatic boundaries in the ANSYS simulation. Hence the following:

(2) $${\left.\frac{\partial T(x,y,t)}{\partial x}\right|}_{y\;=\;-b\;y\;=\;b}\;=\;0$$

(3) $${\left.\frac{\partial T(x,y,t)}{\partial y}\right|}_{y=0}=0$$

where *b* is the half-size of the specimen in the y-direction. Since the laser heat source is modelled as a prescribed surface heat–flux boundary, the top surface is described by a second-kind boundary condition as follows:

(4) $$-k\frac{\partial T}{\partial n}\;=\;q\left(x,y,t\right)$$

where $\frac{\partial T}{\partial n}$ denotes the derivative of temperature taken along the outward surface normal, and $q\left(x,y,t\right)$ is the heat-flux density function. When $q\left(x,y,t\right)$ = 0 no heat-flux load acts on the boundary—the boundary is called adiabatic.

(4) During the laser drilling process, phase change processes such as vaporization, melting, and solidification occur. The latent heat of phase change has a significant impact on the temperature field analysis. Therefore, it is essential to consider the latent heat of phase change in the simulation process. In the ANSYS Mechanical APDL 10.0 software, the latent heat of phase change is incorporated by using the thermal enthalpy material properties (ENTH) [16].

### 2.3. Experimental Parameters

The experiment was designed using a single-factor controlled-variable approach. Pulse width, pulse energy, and pulse count were selected as factors, each evaluated at five levels. The specific factor–level combinations are listed in Table 3.

## 3. Results and Discussion

### 3.1. Simulation Results

Millisecond lasers are classified as long-pulse sources and generate significant thermal effects during drilling. Although this heat facilitates material removal, it also induces thermal damage—including recast layers and micro-cracks—that degrades hole quality. Consequently, analyzing the intrahole temperature field during drilling is essential for minimizing defects and enhancing machining quality.

#### 3.1.1. Effect of Pulse Energy on Hole Quality

To examine the transient temperature field, the pulse count was fixed at 50 and the pulse width at 0.8 ms. The temperature distribution was then simulated for pulse energies of 2.0, 2.2, 2.4, 2.6, and 2.8 J.

**Figure 2:**
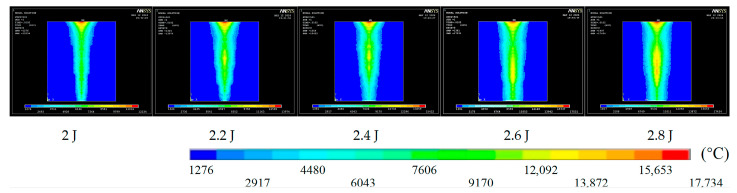
Temperature field cloud for different pulse energies.

**Figure 3:**
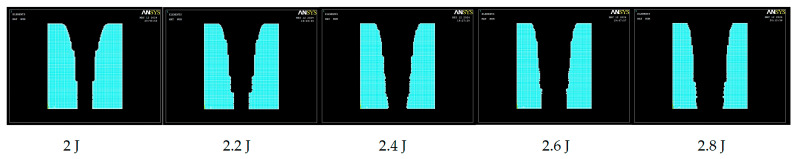
Microporous contours at different pulse widths.

As illustrated in Figure 2, laser irradiation elevates the surface temperature to several tens of thousands of degrees Celsius. The resulting heat propagates radially and toward the exit, creating an intense high-temperature zone. At this energy density, the material melts—and may even vaporize—so the hole gradually forms. Higher pulse energy further intensifies the high-temperature region at the exit. In the simulation, an element birth-and-death technique was applied: elements whose temperature exceeded the melting point were deactivated, whereas cooler elements remained active. The remaining active elements delineate the hole geometry, as illustrated in Figure 3.

Figure 3 shows that increasing pulse energy removes more material and visibly enlarges the exit diameter. The corresponding entrance and exit diameters and taper values are listed in Table 4.

A clear trend is evident in Table 4: as pulse energy is increased, both inlet and outlet diameters of the micro-holes are enlarged, with the enlargement more pronounced at the outlet. Accordingly, hole taper is observed to increase with pulse energy, except for a slight decrease at 2.4 J. This behaviour is attributed to the higher power density, by which high-temperature, high-pressure vapour is generated and expelled from the hole bottom together with molten material. When higher power is applied, more vapour is produced, pressure rises, and additional melt is removed, resulting in a larger hole diameter.

#### 3.1.2. Effect of Pulse Width on Hole Quality

To investigate how pulse width influences micro-hole quality, the pulse energy was fixed at 2.0 J and the pulse count at 50. Temperature-field distributions were then simulated for pulse widths of 0.6, 0.8, 1.0, 1.2, and 1.4 ms.

**Figure 4:**
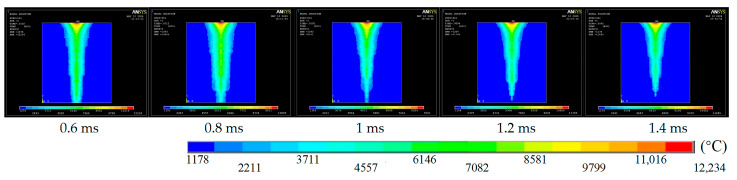
Temperature field cloud for different pulse width.

Figure 4 shows that, as the pulse width is increased, the laser-energy distribution broadens, whereas the temperature near the aperture exit decreases because the peak laser power—and thus the spot energy density—is reduced. When the pulse width reaches 1.2 ms or 1.4 ms, the temperature rise at the exit becomes negligible.

**Figure 5:**
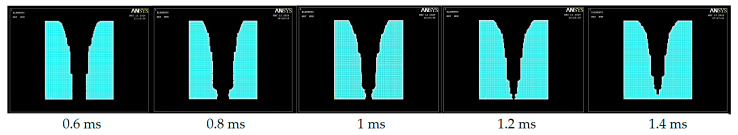
Microporous contours at different pulse widths.

Figure 5 and Table 5 show that, as pulse width increases, progressively less material is removed from the lower surface and hole skewness increases. When the pulse width reaches 1.2 ms, the exit diameter becomes very small, and no through-hole is produced at 1.4 ms. Further increases in pulse width cause both entrance and exit diameters to shrink, the taper to decrease, and eventually prevent breakthrough. This behaviour occurs because lower power density allows heat to accumulate inside the hole; the resulting molten material is not fully expelled, progressively reducing the exit diameter.

#### 3.1.3. Effect of Pulse Number on the Quality of Small Holes

To investigate how pulse count affects micro-via quality, the laser repetition rate was fixed at 50 Hz and the pulse width at 1 ms. Temperature–field distributions were then simulated for pulse counts of 40, 45, 50, 55, and 60.

**Figure 6:**
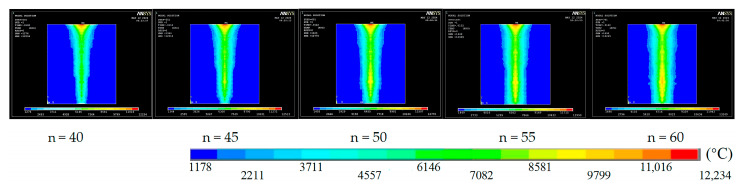
Temperature field cloud for different pulse number.

Figure 6 shows that, upon laser irradiation, the surface temperature rises rapidly and spreads radially around the hole. As the pulse count increases, the high-temperature region expands and heat propagation toward the exit becomes more pronounced.

**Figure 7:**
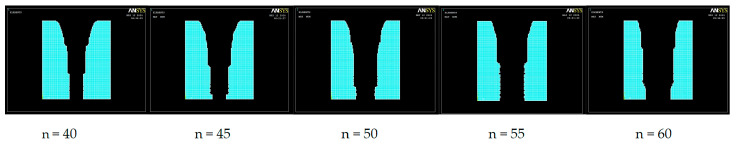
Hole profile formation process with different number of pulses.

Figure 7 and Table 6 indicate that material is removed in a distinct step-like pattern. As pulse count increases, the volume of removed material also rises. The steps deepen and widen, which enlarges both entrance and exit diameters. With further pulses, the entrance diameter continues to grow, whereas the exit diameter first enlarges and then contracts; the taper likewise decreases initially and then increases, exhibiting an overall upward trend.

### 3.2. Experimental Verification and Comparison

**Figure 8:**
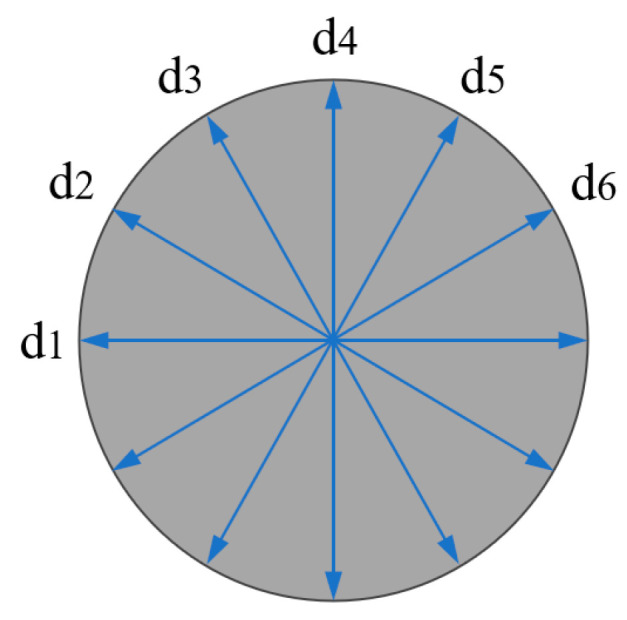
Measurement of hole diameter.

To validate the numerical model, experiments were conducted under the same single-factor conditions as the simulations, In which 3 replicate experiments were performed for each set of parameters to ensure the accuracy of the experimental results. A comparative analysis was subsequently performed between the simulation outputs and the experimental measurements. For diameter measurements, values were taken every 30° around the hole perimeter, and their average was used, as illustrated in Figure 8. Taper was calculated from the measured diameters according to Equation (5).

(5) $$\theta =arctan\left(\frac{d_{\mathrm{entry}}-d_{exit}}{2t}\right)$$

where θ is the hole taper; d_entry_ is the diameter at the hole entrance; d_exit_ is the diameter at the hole exit. Roundness is defined as the ratio of the minimum diameter to the maximum diameter. As shown in Equation (6). Roundness under ideal conditions is 1.

(6) $$C_{entry}=\frac{{\left(d_{min}\right)}_{entry}}{{\left(d_{max}\right)}_{entry}}{,C}_{exit}=\frac{{\left(d_{min}\right)}_{exit}}{{\left(d_{max}\right)}_{exit}}$$

where *C_entry_* is the inlet circularity; *C_exit_* is the outlet circularity; (*d_min_*)*_entry_* is the minimum value of the inlet measured diameter; (*d_min_*)*_exit_* is the minimum value of the outlet measured diameter; (*d_max_*)*_entry_* is the maximum value of the inlet diameter; (*d_max_*)*_exit_* is the maximum value of the inlet diameter.

#### 3.2.1. Effect of Pulse Energy on Hole Quality

To examine how pulse energy influences the entrance and exit diameters and taper of micro-holes, the pulse width was fixed at 0.8 ms and the pulse count at 50. Taper was then evaluated for pulse energies of 2.0, 2.2, 2.4, 2.6, and 2.8 J.

**Figure 9:**
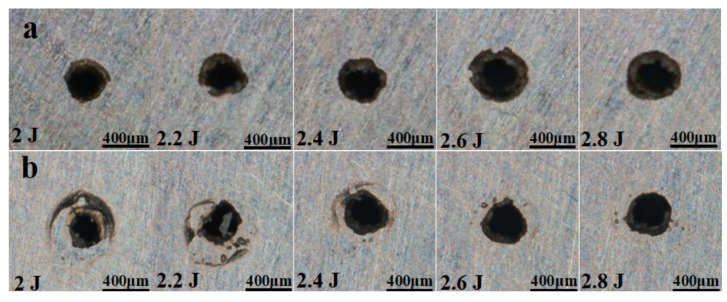
Morphology of micropores at different pulse energies: (**a**) hole inlet and (**b**) hole outlet.

Figure 9a,b show that the aperture diameter gradually increases as pulse energy is raised, owing to the higher spot energy density. The enhanced thermal effect generates additional high-pressure vapour, which expels more molten material and further enlarges the hole diameter.

**Figure 10:**
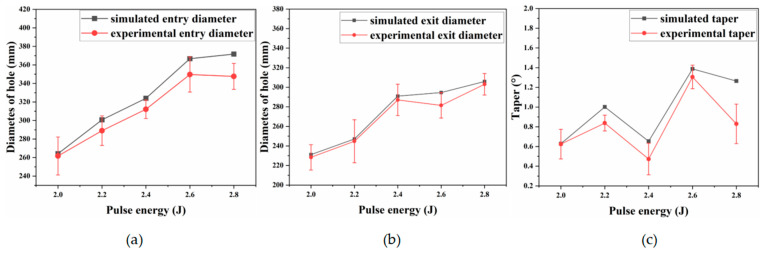
Entrance and exit diameters and taper under different pulse energies: (**a**) entry aperture; (**b**) exit aperture; and (**c**) taper.

**Figure 11:**
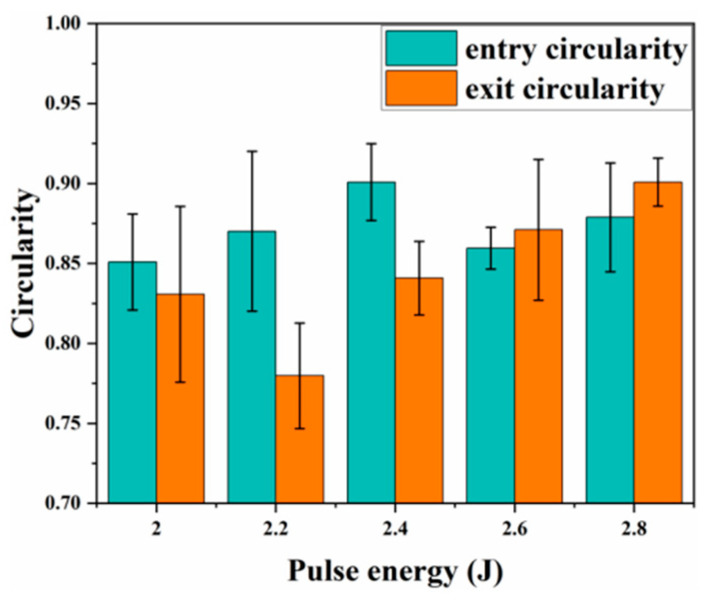
Experimental entry aperture circularity and exit aperture circularity.

Figure 10 shows that the simulated entrance and exit diameters and taper agree closely with the experimental measurements. Figure 11 indicates that inlet and exit circularity increase gradually with pulse energy; at 2.6 J, the exit circularity surpasses the inlet value. This trend arises because higher pulse energy delivers more absorbed energy, which enhances material removal, and the resulting increase in energy density at the exit further improves circularity. However, the greater volume of molten material generated at high energy can flow irregularly, destabilizing exit quality and causing fluctuations in circularity.

#### 3.2.2. Effect of Pulse Width on Hole Quality

To examine how pulse width influences the entrance and exit diameters and taper of micro-holes, the pulse energy was fixed at 2.5 J and the pulse count at 50. Taper was then evaluated for pulse widths of 0.6, 0.8, 1.0, 1.2, and 1.4 ms.

**Figure 12:**
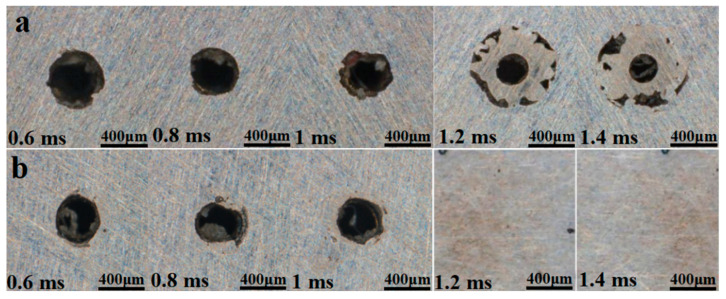
Morphology of micropores at different pulse widths. (**a**) Hole entrance and (**b**) hole exit.

Figure 12a shows that, as pulse width increases, the entrance diameter first decreases and then increases, whereas the exit diameter decreases overall. At short pulse widths, the higher energy density produces stronger thermal effects, enlarging the hole diameter. At longer pulse widths, the lower energy density means that material removal relies mainly on accumulated heat, which diffuses radially and axially from the spot and further enlarges the aperture.

**Figure 13:**
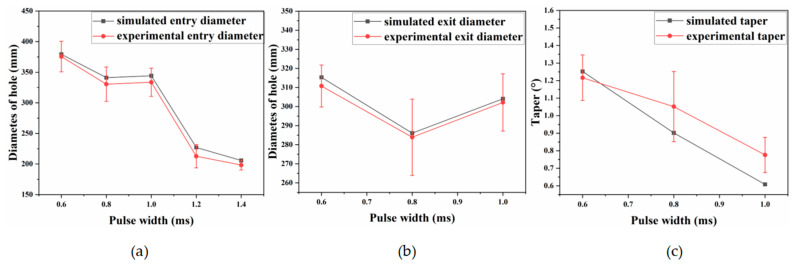
Entrance and exit diameters and taper under different pulse width, (**a**) Entry aperture (**b**) Exit aperture (**c**) Taper.

Figure 13 shows that simulated and experimental values for entrance diameter, exit diameter, and taper follow similar trends, although the simulations consistently overpredict. This discrepancy likely arises because the model neglects plasma formation, beam divergence, and other process phenomena. Between 2.6 and 2.8 J, the simulated entrance diameter deviates from the experimental trend; the error is attributed to the idealized boundary conditions, which omit the effects of assist-gas pressure and melt backflow under gravity.

**Figure 14:**
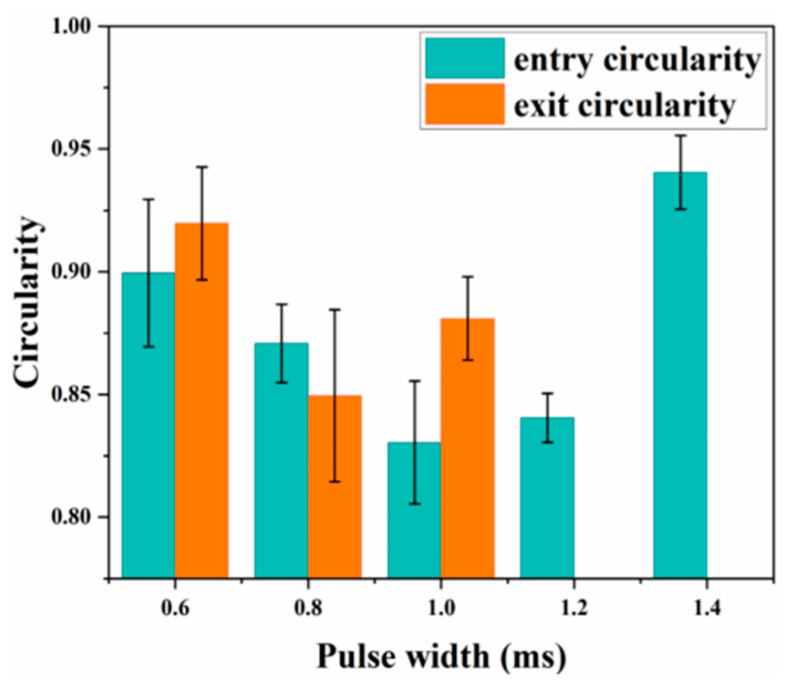
Experimental entry aperture circularity and exit aperture circularity.

Figure 14 indicates that inlet roundness first decreases and then increases with pulse width, whereas exit roundness declines overall. The reduction is explained by the lower spot energy density at longer pulses, which shifts material removal away from the beam centre. At a pulse width of 1.4 ms, inlet roundness rises sharply because no through-hole is formed; the confined vapour pressure expels melt more effectively, increasing the material-removal rate and improving circularity at the entrance.

#### 3.2.3. Effect of Number of Pulses on Hole Quality

To examine how pulse count affects the entrance and exit diameters and taper of micro-holes, the pulse energy was fixed at 2.5 J and the pulse width at 0.8 ms. Taper was then evaluated for pulse counts of 40, 45, 50, 55, and 60.

**Figure 15:**
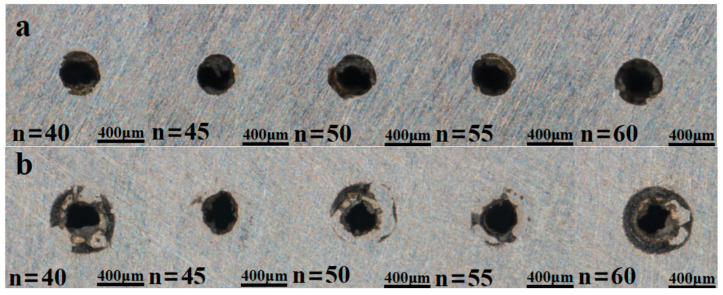
Micro-hole topography at different pulse numbers. (**a**) Hole inlet and (**b**) hole outlet.

Figure 15 shows that the entrance diameter increases with pulse count because additional pulses extend the laser–material interaction time, allowing more material to be ablated.

**Figure 16:**
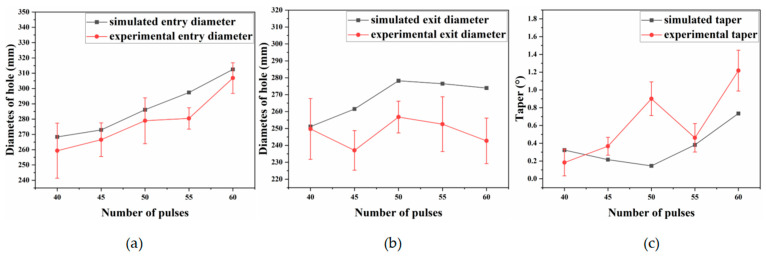
Entrance and exit diameters and taper under different pulse number. (**a**) Entry aperture; (**b**) exit aperture; (**c**) taper.

**Figure 17:**
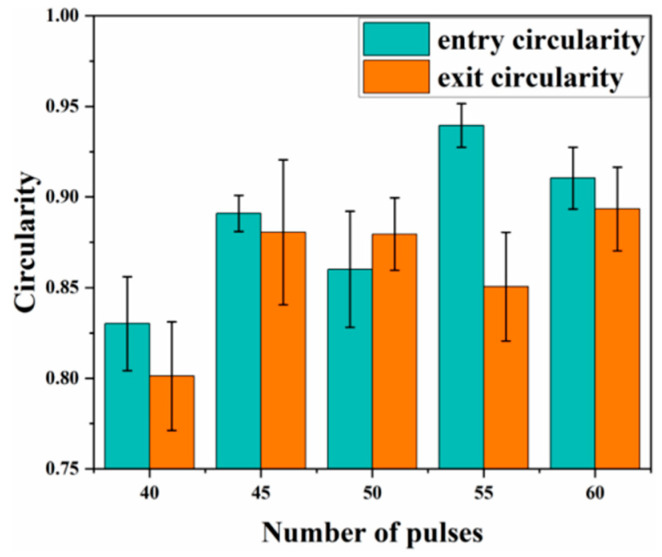
Experimental entry aperture circularity and exit aperture circularity.

Figure 16a,b show that the entrance diameter increases with pulse count; simulated and experimental trends coincide, although the simulated exit diameter deviates more from the measurements. Figure 16c indicates a similar trend for taper, which increases with pulse count; at 45 pulses, the discrepancy between simulation and experiment becomes pronounced. This error probably arises because, in practice, high-temperature, high-pressure vapour flushes molten material along the hole wall, whereas the simulation assumes idealized central ejection. Consequently, the experimental diameters exceed the simulated values. Figure 17 shows that inlet and exit roundness increase irregularly with pulse count. Although a plasma cloud attenuates laser energy during drilling, material removal remains dominated by thermal effects; the high-power millisecond pulses retain sufficient energy to ablate material efficiently, thereby enhancing roundness.

## 4. Conclusions

In this study, titanium alloy TC4 was selected as the research object. ANSYS simulation software was used to simulate changes in the temperature field, pore shape, and pore appearance during the punching process. Subsequently, a millisecond laser percussion punching experiment was conducted. The numerical simulation results were compared with the experimental results. The main conclusions are as follows:

(1) Simulation results show that when the laser acts on the material, it vaporizes and is ejected from the hole, increasing the speed at which molten material is expelled, allowing the laser to reach the bottom more quickly and improving processing efficiency.

(2) Experimental results show that pulse energy and pulse count significantly affect the entrance and exit diameters of the micro-holes, whereas pulse width plays a minor role. When pulse energy rises from 2.0 to 2.8 J, both entrance and exit diameters increase continuously. Pulse count and pulse width exert a stronger influence on taper, whereas pulse energy is less influential. As pulse width increases from 0.6 to 1.4 ms, the taper of the micro-holes increases accordingly. With pulse counts between 40 and 60, entrance and exit diameters as well as taper generally increase. Under otherwise constant conditions, a pulse count of 55 yields the best entrance roundness, whereas a pulse width of 0.6 ms provides the highest exit roundness.

(3) Comparison of numerical simulations with experimental data shows that simulated entrance and exit diameters, as well as taper, are consistently larger than their experimental counterparts. This discrepancy likely arises because the model neglects plasma–cloud formation and beam divergence and assumes a constant energy input to the material.

## Figures and Tables

**Table 1:** Chemical composition of titanium alloy TC4.

Chemical Composition	Content%							
	Ti	Al	V	Fe	C	N	H	O
Ti-6Al-4V	Surplus	5.5~6.8	3.5~4.5	0.3	0.1	0.05	0.015	0.2

**Table 2:** Thermal physical parameters of titanium alloy.

Temperature (°C)	ENTH (1.0 × 10^9^ J·m^−3^)	Specific Heat (1.0 × 10^3^ J·kg^−1^·°C^−1^)	Thermal Conductivity (W·m^−1^·°C^−1^)
25	0.7	0.52	21.9
500	1.81	0.52	21.9
1000	2.99	0.52	21.9
1500	4.16	0.52	21.9
2000	5.33	0.52	21.9

**Table 3:** Single factor experimental plan.

Number	Pulse Energy (J)	Pulse Width (ms)	Number of Pulses (n)
1	22.2, 2.4 2.6, 2.8	0.8	50
2	2.5	0.6, 0.81 1.2, 1.4	50
3	2	0.8	40, 45, 50 55, 60

**Table 4:** Inlet and outlet aperture diameters and taper at different pulse energies.

Material Thickness (mm)	Pulse Energy (J)	Inlet Diameter (µm)	Outlet Diameter (µm)	Taper (deg)
3	2	264	231	0.63
	2.2	300	247	1
	2.4	324	290	0.65
	2.6	367	295	1.38
	2.8	372	306	1.26

**Table 5:** Simulated values of inlet, outlet, and taper at different pulse widths.

Material Thickness (mm)	Pulse Width (ms)	Inlet Diameter (µm)	Outlet Diameter (µm)	Taper (deg)
3	0.6	268	251	0.32
	0.8	273	262	0.21
	1.0	286	278	0.15
	1.2	297	277	0.38
	1.4	312	274	0.73

**Table 6:** Simulated values of inlet, outlet and taper for different number of pulses.

Material Thickness (mm)	Number of Pulses (J)	Inlet Diameter (µm)	Outlet Diameter (µm)	Taper (deg)
3	40	268	251	0.32
	45	273	262	0.21
	50	286	278	0.15
	55	297	277	0.38
	60	312	274	0.73

## Data Availability

The original contributions presented in this study are included in the article material. Further inquiries can be directed to the corresponding author.
